# Collaborative WiFi Fingerprinting Using Sensor-Based Navigation on Smartphones

**DOI:** 10.3390/s150717534

**Published:** 2015-07-20

**Authors:** Peng Zhang, Qile Zhao, You Li, Xiaoji Niu, Yuan Zhuang, Jingnan Liu

**Affiliations:** 1 GNSS Research Center, Wuhan University, No.129 Luoyu Road, Wuhan 430079, China; E-Mails: fenix@whu.edu.cn (P.Z.); liyou@whu.edu.cn (Y.L.); xjniu@whu.edu.cn (X.N.); jnliu@whu.edu.cn (J.L.); 2 Department of Geomatics Engineering, University of Calgary, Calgary, AB T2N1N4, Canada; E-Mail: zhy.0908@gmail.com

**Keywords:** WiFi, indoor positioning, MEMS sensors, training, PDR

## Abstract

This paper presents a method that trains the WiFi fingerprint database using sensor-based navigation solutions. Since micro-electromechanical systems (MEMS) sensors provide only a short-term accuracy but suffer from the accuracy degradation with time, we restrict the time length of available indoor navigation trajectories, and conduct post-processing to improve the sensor-based navigation solution. Different middle-term navigation trajectories that move in and out of an indoor area are combined to make up the database. Furthermore, we evaluate the effect of WiFi database shifts on WiFi fingerprinting using the database generated by the proposed method. Results show that the fingerprinting errors will not increase linearly according to database (DB) errors in smartphone-based WiFi fingerprinting applications.

## 1. Introduction

Mobile location-based services (LBS) are attracting the attention of many mobile device companies due to their potential applications in a wide range of personalized services [1]. As a core technology of LBS, navigation (*i.e.*, determination of the position, velocity, and attitude of the mobile device) is evidently vital. A highly demanding issue is to provide trustable navigation solutions in real time, since mobile devices are carried by users almost anywhere and anytime [2].

While Global Navigation Satellite Systems (GNSS) based outdoor navigation has greatly advanced over the past few decades, positioning and navigation in indoor scenarios and deep urban areas are still an open issue [3]. The challenges include unavailable or degraded GNSS signals, complex indoor environments, the necessity of using low-grade devices, *etc.* To navigate in indoor or urban areas, various indoor positioning technologies based on Radio Frequency (RF) signals have been broadly researched, such as Wireless Local Area Net (WLAN, also known as Wi-Fi) [4], cellular networks [5], Bluetooth [6], Radio Frequency identification (RFID) tags [7], ZigBee [8], Ultra Wideband Beacons (UWB) [9], high-sensitivity GNSS [10], and pseudo-satellites (pseudolites, also known as Locata) [11]. These RF-based technologies can provide a long-term accurate absolute position, but require the creation and maintenance of a network [12]. WiFi (WLAN based on 802.11 standards) does not need a specific network, since there are WiFi infrastructures in public areas such as malls, airports, and universities. Considering the fact that the WiFi receivers commonly exist in consumer devices such as smartphones, it is feasible to implement WiFi positioning in these public areas. The common WiFi positioning approaches include triangulation [13], fingerprinting [14,15,16,17,18], and their combination [19]. Triangulation is a method that determines the relative positions of objects using the geometry of triangles; therefore, a signal propagation model is needed to convert the received signal strength (RSS) to calculate the distance between WiFi access points (APs) and user devices [20]. However, it is difficult to obtain an accurate signal propagation model in indoor environments, since transmitted signals can suffer obstructions and reflections [21]. To mitigate this issue, several enhanced models considering multipath effects [22] or Rayleigh fading effects [23] have been studied. WiFi fingerprinting approaches based on WiFi RSS have gained a large amount of attention, as they can provide a relatively high indoor positioning accuracy in a multipath indoor environment [24]. Fingerprinting is commonly achieved in two steps (phases): the offline training (pre-survey) step and the online positioning step. The training phase is conducted to build or update a <RSS, location> database (DB) that consists of a set of reference points (RPs) with known coordinates and the RSS from available WiFi APs, while the positioning step is implemented to finding the closest match between the features of the RSS and those stored in the DB. The main barrier for the broad adoption of WiFi fingerprinting is that most current DB training methods are tedious and labor-intensive [25]. Another challenge is the non-stationarity of WiFi signal distribution, which can be attributed to radio signal propagation effects induced by environment changes [20,26,27,28]. Therefore, the training phase needs to be rerun periodically to keep the system up-to-date [24].

Different WiFi DB training approaches have been researched due to various requirements. The research [29,30] have proposed the idea of a metropolitan-scale WiFi localization based on vehicles with equipment such as GNSS receivers and high-end inertial navigation systems. This method is highly efficient, but is mainly used in outdoor urban areas. To train indoor WiFi DBs, the first approach is to survey at every RP and record their fingerprints. This method can also improve DB reliability by averaging the RSS at each RP [31] and even providing a coarse estimate of orientation [20,32]. However, it is time- and labor-consuming when dense RPs are selected to cover an entire area of interest, and a surveyor needs to mark the position of all RPs (labels) manually [33]. The training phase can take up to several hours even for a small building [34].

To minimize the number of labels needed for training, another approach is used based on landmarks (*i.e.*, points with known coordinates) or floor plans (*i.e.*, the true position of corners and intersections, and the true orientation of corridors), and constant-speed assumption. To implement this method, a surveyor has to walk with constant speed along each link between landmarks, such as corners or intersections, over the known path. The position of landmarks can be determined by marking them manually on a digital map, while the position of other RPs on the links between landmarks can be calculated by the arrival time and the distance between two landmarks. This approach is significantly more time-effective than measuring the positon of all RPs on the digital map; however, it requires the user to walk straight with a constant speed between landmarks. To break the limitation of the constant-speed assumption, scholars generate the WiFi fingerprint DB between landmarks by leveraging a dead-reckoning solution from sensors [35]. Sensors can provide a step-based dead-reckoning approach, which detects steps, predicts their length and direction based on sensor readings [24].

There is also literature on the removal of the training process by updating the WiFi DB automatically while navigating based on sensors. WiFi SLAM (simultaneous location and mapping) is an advanced algorithm which trains WiFi DB while navigating [36]. The limitation of the SLAM algorithm is the increased computational cost as the scenarios become larger [37]. There are other approaches based on crowd-sourcing. The research [38] estimates the location of WiFi APs or other radio beacons using pedestrian dead-reckoning with high-quality foot-mounted IMUs, while [34,39,40,41] propose similar systems or approaches using handheld smartphones. Based on this idea, it is possible for mobile users to collect WiFi fingerprints automatically in daily life by conducting sensor-based navigation. The crowd-sourcing approaches have the potential to provide daily-life navigation solutions due to the increasing popularity of Micro-electromechanical Systems (MEMS) sensor in consumer electronics [42,43]. The shortcoming is that MEMS sensor errors change with time and are significantly dependent on environmental factors such as temperature [44]. Therefore, MEMS inertial sensors provide only short-term accuracy but suffer from accuracy degradation over time [45]. Although the errors of horizontal attitudes (*i.e.*, the roll and pitch) can be controlled by accelerometer measurements, the heading error will grow when there is no additional information from other sensors or techniques [46]. Magnetometers can assist the heading estimation by sensing the Earth’s magnetic field; however, the local magnetic field is susceptible to interference from man-made infrastructures when the user enters urban or indoor environments [47], which makes magnetometer measurements unreliable. Therefore, MEMS sensors have to be integrated with other positioning technologies to provide reliable long-term navigation solutions.

In this paper, we propose an approach that utilizes similar ideas as [34,39,40], *i.e.*, training the WiFi DB using the navigation data from the users, and utilizing different strategies to control sensor-based navigation errors and in turn control the drifts of the generated WiFi DBs. First, we use a strategy that combines different navigation trajectories that move in and out of a building to make up the DB; also, we restrict the time length of available indoor navigation trajectories, *i.e.*, the trajectories from the last epoch that receives GNSS signal before entering an indoor area to the first epoch that receives GNSS signal after walking out of the indoor area. To be specific, we only use the navigation data which is shorter than a time threshold (e.g., 10 min). This strategy can help control the sensor-based navigation errors, but will exclude the majority of navigation trajectories. However, since there will be massive pedestrian navigation data in real-world application, even a small number of reliable navigation trajectories will be enough to generate the WiFi DB; also, it is necessary to use only the most valuable data and exclude others. Moreover, sometimes aiding sources such as landmarks can update the navigation solution, which divide long-term trajectories into short-term trajectories.

Another strategy is implemented based on the fact that users will always walk both into and out of an indoor area. Therefore, we conduct post-processing (smoothing) to improve the sensor-based navigation solution, since there are accurate GNSS position updates on the start and end points of indoor trajectories. Post-processing is common for survey applications such as mobile mapping [48,49], but is used relatively little in real-time navigation [50]. To train the WiFi DB, post-processing is both affordable and worthwhile. Test results show that the navigation solutions can be significantly improved through post-processing, but still suffer from errors during the middle part of the trajectories, which will in turn cause DB drifts.

Furthermore, we evaluate the effect of DB shifts on WiFi fingerprinting. Therefore, the difference between the WiFi fingerprinting results with the proposed DB (*i.e.*, the DB generated by the proposed approach) and the reference DB was not significant when comparing with the WiFi fingerprinting errors with smartphones. Therefore, although constructing a high-quality DB is vital since fingerprinting accuracy highly depends on DB quality [31], the fingerprinting errors will not increase linearly according to DB errors because there are other error sources in smartphone-based WiFi fingerprinting.

This paper is organized as follows: Section 2 provides the methodology of proposed sensor-based navigation methods, including attitude determination, pedestrian dead-reckoning (PDR), and smoothing. Section 3 explains WiFi fingerprinting and focuses on training the WiFi DB; Section 4 outlines the tests and results; and Section 5 draws the conclusions.

## 2. Sensor-Based Navigation

**Figure 1:**
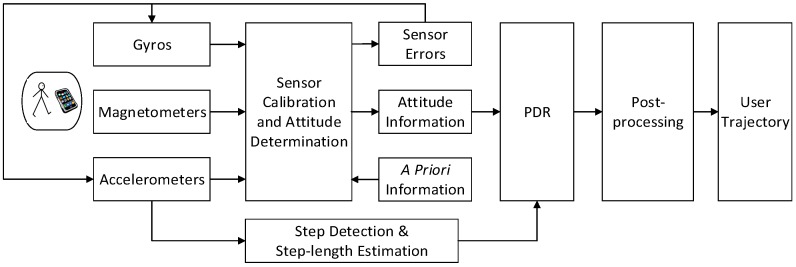
Main components of sensor-based navigation algorithm.

The sensor-based navigation algorithm consists of three modules: multi-sensor based attitude determination, position tracking, and post-processing. The main components of the proposed algorithm are shown in Figure 1. The following will introduce these modules separately.

### 2.1. Multi-Sensor Based Attitude Determination

In this part, we use the inertial navigation systems (INS) mechanization to calculate continuous attitude angles, and utilize the information from multiple sensors and *a priori* information as updates to estimate the attitude errors through an attitude determination Kalman filter. The details of INS mechanization has been well described in [48]. The following will describe the Kalman filter models, including the system model and the measurement models.

#### 2.1.1. Kalman Filter System Model

A simplifies from of the error model detailed in [48] is applied as the continuous state equation in the Kalman filter [51]

(1) $$\left[\begin{array}{c} \delta {\dot{r}}^{n}\\ \delta {\dot{v}}^{n}\\ \dot{\psi } \end{array}\right]=\left[\begin{array}{c} -\omega_{en}^{n}\times \delta r^{n}+\delta v^{n}\\ -(2\omega_{ie}^{n}+\omega_{en}^{n})\times \delta v^{n}+f^{n}\times \psi +C_{b}^{n}\delta f^{b}\\ -(\omega_{ie}^{n}+\omega_{ec}^{n})\times \psi -C_{b}^{n}\delta \omega_{ib}^{b} \end{array}\right]$$

where $\delta r^{n}$, $\delta v^{n}$ and Ψ are the position error, the velocity error, and the attitude error. $C_{b}^{n}$ is the Direction Cosine Matrix from b-frame (*i.e.*, the body frame) to n-frame (*i.e.*, the navigation frame). $C_{b}^{n}$ is the specific force vector projected to n-frame, and $\omega_{ie}^{n}$ and $\omega_{en}^{n}$ represent the angular velocity of the earth and the angular rate vector of n-frame with respect to e-frame (*i.e.*, the Earth frame), both projected to n-frame. The symbol “×” denotes cross product of two vectors. $\delta {\text{f}}^{b}$ and $\delta \omega_{ib}^{b}$ are the accelerometer error and gyro error vectors.

#### 2.1.2. Kalman Filter Measurement Models

We use multiple kinds of constraints to build the measurement model to enhance the attitude determination. These constraints include the pseudo-observations and the measurements from magnetometers and accelerometer.

The pseudo-position and pseudo-velocity observations are proposed based on the fact that the range of the position and linear velocity of the IMU are within a limited scope [51]. They can be used to compose the measurement vectors of the Kalman filter, that is

(2) $${\hat{r}}^{n}-{\hat{v}}_{0}^{n}=\delta r^{n}+n$$

with ${\hat{v}}_{0}^{n}=\text{constant}$ and

(3) $${\hat{v}}^{n}=\delta v^{n}+n_{v}$$

where ${\hat{r}}^{n}$ and ${\hat{v}}^{n}$ are INS-derived position and velocity vectors; ${\hat{v}}_{0}^{n}$ is the observation vector of the proposed pseudo-position; $\delta r^{n}$ and $\delta v^{n}$ are the position errors and velocity errors. $n_{r}$ and $n_{v}$ are the measurement noises (*i.e.*, the inaccuracy) of pseudo-position and pseudo-velocity. The pseudo-velocity is directly set as zero; the pseudo-position can be set as a random constant value and this will not influence the estimation of attitude and gyro errors [51].

Accelerometers and magnetometers are well used to update attitude estimation in many applications of attitude and heading reference systems (AHRS) [52]. The details of using the accelerometer and magnetometer measurements can refer to [53]. The measurement uncertainties on the magnetometer measurements are different from that on the accelerometer signals. The latter is commonly high-frequency and alternating; however, the perturbation on the magnetometer measurements is low-frequency because the local magnetic field (LMF) changes due to the existence of external magnetic bodies such as man-made infrastructures. Among various kinds of perturbations to LMF, a common type is that both the direction and the strength of LMF are changed, but the change is stable during short periods. This period during which the LMF is stable is called quasi-static magnetic field (QSMF) period. We only use the magnetometer measurements in QSMF environment, which follows [47].

#### 2.1.3. Initial Alignment

The initial direction cosine matrix ${\hat{C}}_{b}^{n}(t_{0})$ can be determined by using the accelerometer and magnetometer measurements as [54]

(4) $${\tilde{C}}_{b}^{n}={\left({\left[\begin{array}{ccc} f^{n} & m^{n} & l^{n} \end{array}\right]}^{T}\right)}^{-1}{\left[\begin{array}{ccc} {\tilde{f}}^{b} & {\tilde{m}}^{b} & {\tilde{l}}^{b} \end{array}\right]}^{T}$$

where $f^{n}$ and $m^{n}$ are the specific force and local magnetic field in the navigation frame, $l^{n}=f^{n}\times m^{n}$; ${\tilde{f}}^{b}$ and ${\tilde{m}}^{b}$ are the accelerometer and magnetometer measurements, and ${\tilde{l}}^{b}={\tilde{f}}^{b}\times {\tilde{m}}^{b}$. Since the purpose of this paper is to use sensor-based solution to build the WiFi DB, we start data collection from outdoor environment, where GNSS positioning results are used to provide initial position and heading.

### 2.2. Position Tracking

#### 2.2.1. Pedestrian Dead-Reckoning

PDR is the relative means of determining of a new position from the previous known position using current heading and step length information [55]. The coordinates $\left(\varphi_{k+1},\;\;\lambda_{k+1}\right)$ of a new position with respect to a previously known position $\left(\varphi_{k},\;\;\lambda_{k}\right)$ can be computed as follows:

(5) $$\left[\begin{array}{c} \varphi_{k+1}\\ \lambda_{k+1} \end{array}\right]=\left[\begin{array}{c} \varphi_{k}+(s_{k}\cos\psi_{k-1}+w_{N})/(R_{m}+h)\\ \lambda_{k}+(s_{k}\sin\psi_{k-1}+w_{E})/[(R_{n}+h)cos\varphi_{k}] \end{array}\right]$$

where φ, λ, ψ, and s are the latitude, longitude, heading and step length, $R_{m}$ and $R_{n}$ are the radius of curvature of meridian and curvature in the prime vertical, and h is the altitude. The subscript *k* and *k* + 1 indicate the count of steps.

In practice, the linear error Model (6) is used to implement the PDR algorithm. This is because when other information such as GNSS or WiFi is available, it can be used to update the PDR through a position tracking Kalman filter. The Kalman filter system model is

(6) $${\hat{x}}_{k+1}=\Phi_{k}{\text{x}}_{k}+w_{k}$$

with

(7) $${\hat{x}}_{k}={\left[\begin{array}{ccccc} \delta \varphi_{k} & \delta \lambda_{k} & \delta \psi_{k} & \delta s_{k} & b_{k} \end{array}\right]}^{T}$$

(8) $$\Phi_{k}=\left[\begin{array}{ccccc} 1 & 0 & -s_{k}\sin\psi_{k}/(R_{m}+h) & \cos\psi_{k}/(R_{m}+h) & 0\\ 0 & 1 & s_{k}\cos\psi_{k}/[(R_{n}+h)cos\varphi_{k}] & \sin\psi_{k}/[(R_{n}+h)cos\varphi_{k}] & 0\\ 0 & 0 & 1 & 0 & \Delta t\\ 0 & 0 & 0 & 1 & 0\\ 0 & 0 & 0 & 0 & 1 \end{array}\right]$$

(9) $$w_{k}={\left[\begin{array}{ccccc} w_{N,k}/(R_{m}+h) & w_{E,k}/[(R_{n}+h)cos\varphi ] & w_{\psi } & w_{s} & w_{b} \end{array}\right]}^{T}$$

where $\hat{x}$ is the state vector, $\Phi_{k}$ is the transition matrix, and $w_{k}$ is the noise vector. δφ, δλ, δψ, δs, and b are the errors of latitude, longitude, heading, step length, and the vertical gyro bias component.

#### 2.2.2. Step Length Model

Step detection and step length estimation have been well introduced in [56]. The basic idea is that the acceleration shows a cycle pattern responding to every step when a pedestrian is walking. The specific force signals are smoothed to reduce the influence of noise by

(10) $${\bar{f}}_{k}=\sum_{i=k-L}^{k+L}f_{i}$$

where (2*L* + 1) is the length of smooth array. The smoothed specific force is computed and then checked periodically in a sliding window. If it is a peak and the magnitude is bigger than a threshold, a new step is detected.

The step length varies with walk velocity, terrain scope, *etc.* It can be related to the step frequency with a linear model [57]

(11) $$s_{k}=Af_{k}+B+w_{s}$$

with $f_{k}=1/(t_{k}-t_{k-1})$. $f_{k}$ is the walking frequency from time $t_{k-1}$ to $t_{k}$. A and B are coefficients that can be trained, $w_{s}$ is noise.

### 2.3. Post-Processing

To build WiFi DB, it is affordable and worthwhile to employ post-processing to provide a better navigation solution. In general, smoothing can be performed by combining the forward and backward filter solution as follows [48]:

(12) $${\hat{x}}_{sm,k}={\text{P}}_{sm,k}({\text{P}}_{f,k}^{-1}{\text{x}}_{f,k}+{\text{P}}_{b,k}^{-1}{\hat{x}}_{b,k})$$

(13) $$P_{sm,k}={\left(P_{f,k}^{-1}+P_{b,k}^{-1}\right)}^{-1}$$

where subscripts ‘*f*’, ‘*b*’, and ‘*sm*’ denote the forward, backward, and smoothed solutions, respectively. $\hat{x}$ is the estimated state vector, and P represents the covariance matrix of $\hat{x}$. In this research, $\hat{x}=\left[\begin{array}{ccccc} \varphi & \lambda & \psi & s & b \end{array}\right]$. $P_{f}$ and $P_{b}$ are predicted in the forward and backward process by [58]

(14) $$P_{k+1}=\Phi_{k}P_{k+1}\Phi_{k}^{T}+Q_{k}$$

where Q is the covariance matrices of system noise sequence vector.

When other information such as GNSS or WiFi is available, the matrix P can be updated through

(15) $$P_{k+1}^{+}=\left(I-K_{k+1}H_{k}\right)P_{k+1}^{-1}$$

with

(16) $$K_{k+1}=P_{k+1}H_{k}^{T}\left(H_{k}P_{k+1}H_{k}^{T}\right)+R_{k+1}$$

where K is the Kalman gain matrix, H is the design matrix for measurements, and R is the covariance matrices of measurement error vector.

## 3. WiFi Fingerprinting

**Figure 2:**
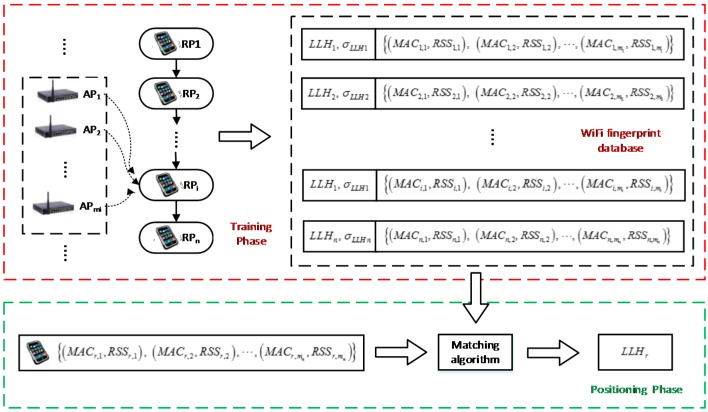
Procedure of WiFi fingerprinting.

WiFi fingerprinting is the widely used positioning approach based on WiFi RSS. The main advantage is that it does not rely on known WiFi AP position and signal propagation environment. WiFi fingerprinting consists of two phases: offline training phase and online positioning phase. The procedure of WiFi fingerprinting is shown in Figure 2 [59].

### 3.1. Training Phase

The purpose of training phase is to build or update a <location, RSS> DB that consists of a set of RPs with known coordinates and RSS from available WiFi APs. To generate DB, enough RPs should be selected to cover the whole area of interest. Then, the RSS from available APs are collected at every RP. The procedure for training is illustrated in the red dashed box in Figure 2. The fingerprint information at the *i*-th RP is recorded as

(17) $$F_{i}=\left\{LLH_{i},\;\sigma_{LLHi},\;\left(MAC_{i,1},RSS_{i,1}\right),\;\;\left(MAC_{i,2},RSS_{i,2}\right),\;\cdots ,\;\left(MAC_{i,m_{i}},RSS_{i,m_{i}}\right)\right\}$$

where $F_{i}$ is fingerprint information at $RP_{i}$, $LLH_{i}$ and $\sigma_{LLHi}$ are the coordinate of $RP_{i}$ and its accuracy, $MAC_{i,j}$ and $RSS_{i,j}$ are the MAC address and RSS of the *j*-th AP received at $RP_{i}$, and $m_{i}$ is the number of available APs at $RP_{i}$.

In this paper, we use the continuous sensor-based navigation solution from the method described in Section 2 to obtain the position of RPs. Therefore, it is possible to train DB with daily-life navigation data from users, instead of performing a separate training phase. This is similar as [34,39,40].

### 3.2. Positioning Phase

In the positioning phase, the user location is estimated by comparing the RSS information with that stored in the DB. The procedure for positioning is illustrated in the green dashed box in Figure 2. Several approaches have been proposed for estimating the user location using RSS measurements, such as the nearest neighbor (NN) approach and the probabilistic estimation method [60]. The NN method selects the RP which has the minimal signal strength distance as the user’s estimated position which is calculated as follows [31]:

(18) $$d_{i}=\sqrt{\sum_{j=1}^{n_{i}}\left|SS_{rec,l_{u}}^{j}-SS_{DB,i}^{j}\right|},\;\;i\in I_{RP}$$

where, $d_{i}$ is the signal strength distance at $RP_{i}$ in the DB, $SS_{rec,lu}$ is the measured RSS vector at $l_{u}$, $SS_{DB,i}$ is the RSS vector at $RP_{i}$, $n_{i}$ is the number of WiFi signals received at $RP_{i}$, and $I_{RP}$ is the location index set of RPs in the DB. Then the coordinates of $RP_{i}$ which satisfies the condition $d_{i*}=\min\left(d_{i}|i\in I_{RP}\right)$ is determined as the position estimation of $l_{u}$.

We use a multi-level quality control mechanism to optimize WiFi positioning. The first is the measurement level, where a threshold $Th_{RSS}$ is set to filtering out APs with weak RSS. The second level is based on the minimal signal strength distance. If the minimal signal strength distance at a certain epoch is larger than the given threshold $Th_{d}$, we will not use the fingerprinting results at this epoch because probably the current user location has not been stored as a RP in the DB.

Furthermore, to mitigate the impact of blunder and get a more reliable positioning result, the *k*-NN estimation technique is considered [59], which estimates the position according to the *k* RPs that have the smallest distances. The position estimate is obtained by a weighed sum of the positions of the nearest RPs by

(19) $$\hat{r}=\sum_{i=1}^{k}\frac{c_{i}}{C}r_{i}$$

where $c_{i}=1/d_{i}$, $C=\sum_{i=1}^{k}c_{i}$, $r_{i}$ is the position of the *i*-th nearest RP, $\hat{r}$ is the estimated position.

## 4. Tests and Results

Walking tests were conducted in two indoor environments: the Energy, Environment, and Experiential learning (EEEL) building, in which the average weighted AP number (as defined in Section 4.1.2) at one point was over 15; and the Engineer building (ENB), in which the average weighted AP number was nearly seven. The tests were performed with a Samsung Galaxy 4 smartphone. A designed software was used to simultaneously receive the data from sensors, WiFi, and GNSS. The sample rate of sensors, WiFi, and GNSS data were set as 20 Hz, 1 Hz, and 1 Hz, respectively. Even though our attitude estimation algorithm can dealing with different scenarios such as handheld, ear, dangling, pocket, and backpack [61], we conducted the tests in this paper with only handheld mode to focus on investigating WiFi positioning.

### 4.1. Tests at EEEL

**Figure 3:**
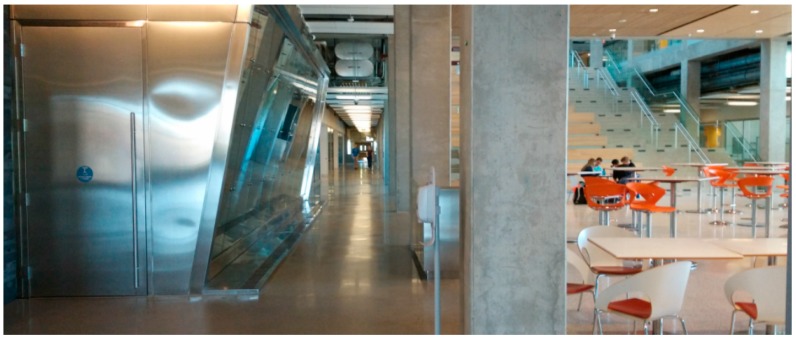
Indoor test environment at EEEL.

EEEL is a relatively new building with well-equipped infrastructures. There are metallic infrastructures inside the building, which may influent the propagation of WiFi signals. Figure 3 shows the indoor test environment. This building has a main corridor which is 3 m wide, and a lobby which is about 30 × 30 m^2^. The test area at EEEL was approximately 120 × 40 m^2^.

#### 4.1.1. Trajectories for Building DB

**Figure 4:**
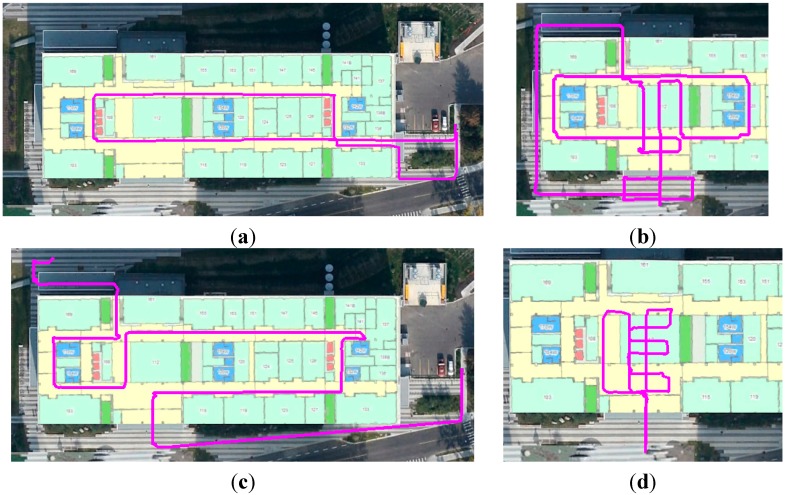
Different trajectories used to generate WiFi DB (**a**) Trajectory 1; (**b**) Trajectory 2; (**c**) Trajectory 3; (**d**) Trajectory 4.

In this test, we generated the WiFi fingerprint DB inside the EEEL building using four different sensors stand-alone navigation trajectories. The true trajectories are shown in Figure 4. Each trajectory lasted for 5–10 min. Both the starting and ending points of each trajectory were in the outdoor environment, where the initial position was provided by GNSS.

#### 4.1.2. Building WiFi DB Using Sensor-Based Navigation Solutions

**Figure 5:**
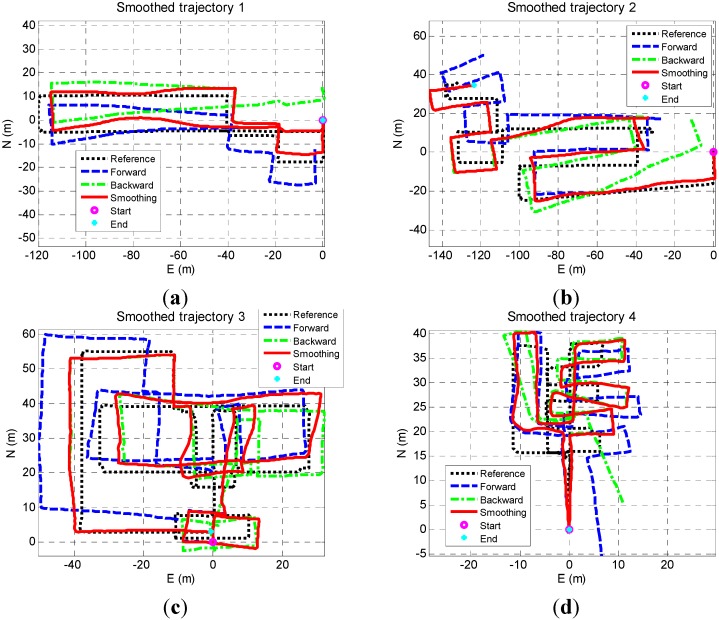
Sensor-based navigation solutions (**a**) Trajectory 1; (**b**) Trajectory 2; (**c**) Trajectory 3; (**d**) Trajectory 4.

Figure 5a–d shows the sensor-based navigation solutions in a local geographic frame. In each figure, the blue dash line, green dashed line, red solid line, and black dotted line are the forward, backward, smoothed result, and the reference. The reference is provided by correcting the PDR solution aided by floor plan (*i.e.*, the true position of corners and intersections, and the true orientation of corridors). The floor plan information was obtained from Google Earth. The start and end points indicates the starting points of forward and backward PDR, respectively.

**Figure 6:**
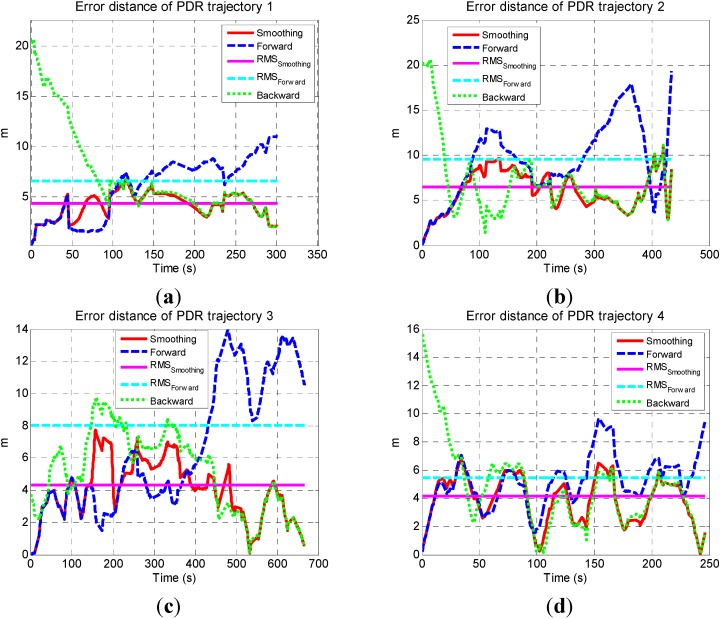
Error distances of sensor-based navigation solutions (**a**) Trajectory 1; (**b**) Trajectory 2; (**c**) Trajectory 3; (**d**) Trajectory 4.

Figure 5 shows that both forward and backward PDR trajectories had a similar shape with the reference and are accurate at the beginning, but suffered from long-term drifts. The drifts were caused by both heading and step length errors, which are the issues inherent in the sensors-only navigation algorithm. The smoothed trajectory significantly got closed to the reference at beginning and ending periods since forward and backward PDR solutions were accurate and had high weight at beginning and ending periods, respectively. To make the errors clear, the error distances (*i.e.*, the distance between estimated user position and the corresponding true position) of these solutions are illustrated in Figure 6a–d. In each figure, the blue dashed line, green dotted line, and red solid line represent the error distances of forward, backward, and smoothing result, respectively. The magenta solid line and cyan dashed line indicate the RMS values of forward and smoothed results.

The smoothed results were accurate at the beginning and ending parts of each trajectory, and had more drifts in the middle, which meets the characteristics of smoothing algorithm. By comprehensive using the data during the whole navigation process, the smoothed results were more accurate than the forward. The max and RMS values of the errors in forward and smoothed results are shown in Table 1. Each element in the final row is the RMS of the values of four trajectories in the corresponding column.

**Table 1:** Statistical values of sensor-based navigation errors.

Trajectory	Errors in Forward Results		Errors in Smoothed Results		RMS Reduction
	Max	RMS	Max	RMS	
1	11.0	6.6	6.7	4.3	34.9%
2	19.3	9.5	11.1	6.5	31.6%
3	14.0	8.0	7.7	4.3	46.3%
4	9.6	5.5	7.0	4.1	25.5%
RMS	16.1	8.7	9.6	5.7	34.5%

**Figure 7:**
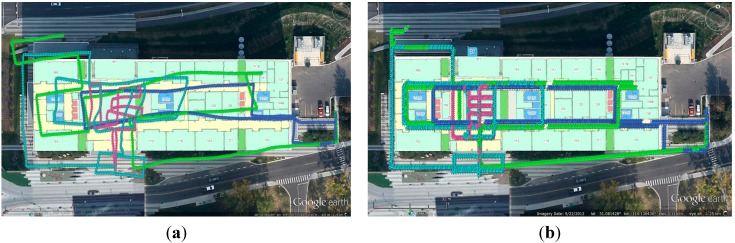
The DB built through proposed method (**a**), and the reference DB built by using the conventional floor plan aided training approach (**b**).

The max and RMS values of error distance were generally reduced from 16.1 m and 8.7 m to 9.6 m and 5.7 m after smoothing, with a reduction of 34.5%. Then, the smoothed navigation solutions were used to build the WiFi fingerprint DB. The DB built through the proposed method (which will be denoted as “the proposed DB” for short) is shown in Figure 7a. To make a comparison, a reference DB was built by using the conventional floor plan aided training approach (which will be denoted as “the reference DB” for short) and shown in Figure 7b. It is clear that the proposed DB has some shifts when comparing with the true path, while the reference DB fits the true path. We will evaluate the effect of such DB shift on WiFi positioning errors by WiFi positioning tests in the next subsection.

**Figure 8:**
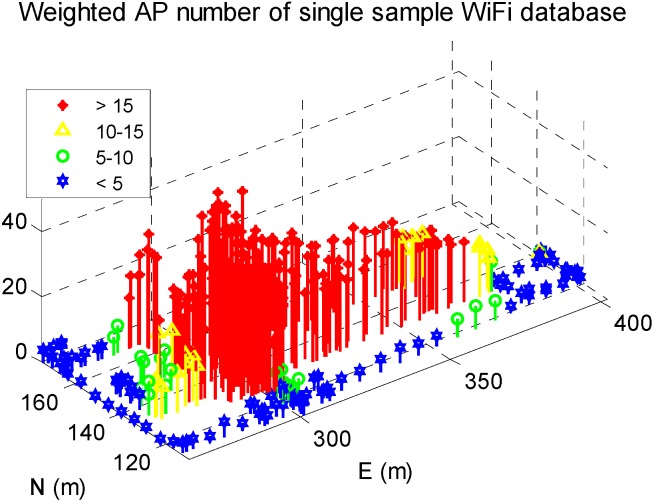
WiFi signal distribution in reference DB.

In addition, WiFi signal distribution in the reference DB was shown in Figure 8. The *x*- and *y*-axis indicate the length in the west-east and south-north directions, and the *z*-axis show the weighted AP number, which is calculated by

(20) $$WAP_{i}=\sum_{j=1}^{n_{i}}a_{i,j},\;\;i\in I_{RP}$$

where $WAP_{i}$ is the weighted AP number at $RP_{i}$ in the DB, $n_{i}$ is the number of WiFi signals received at $RP_{i}$, $I_{RP}$ is the location index set of RPs in the DB. The value of $a_{i,j}$ is determine according to $RSS_{i,j}$ (*i.e.*, the RSS of $AP_{j}$ at $RP_{i}$) by the following rule: if $RSS_{i,j}$ > −60 dBm, $a_{i,j}$ = 1; else if −70 dBm < $RSS_{i,j}$ < −60 dBm, $a_{i,j}$ = 0.75; else if −85 dBm < $RSS_{i,j}$ < −70 dBm, $a_{i,j}$ = 0.25; else if $RSS_{i,j}$ < −85 dBm, $a_{i,j}$ = 0. Compared with Figure 7b, the available WiFi signals were abundant in the middle area of EEEL, lesser but still enough in the marginal indoor areas, and even less in outdoor areas.

#### 4.1.3. WiFi Fingerprinting Using Generated DB

**Figure 9:**
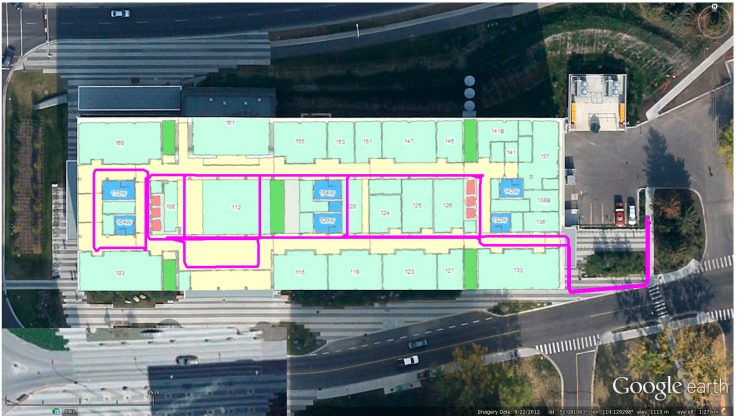
The trajectory used to test WiFi fingerprinting.

The WiFi fingerprinting performance with both the proposed DB and the reference DB was tested by a separate navigation trajectory in the EEEL building, to evaluate the effect of DB shifts on WiFi positioning errors. The true trajectory is shown in Figure 9.

**Figure 10:**
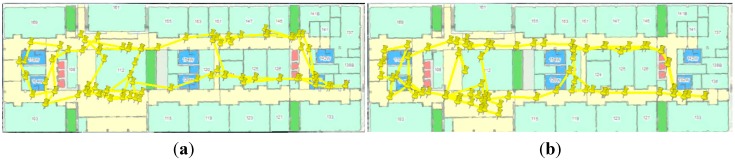
WiFi fingerprinting result using proposed DB (**a**) and that using reference DB (**b**).

The parameters were set as $Th_{RSS}=-85\;\text{dBm}$ and $Th_{d}=200\;\text{dBm}$ in WiFi data processing. That is, only the RSS stronger than −85 dBm were used, and the WiFi fingerprinting results was used only when the minimal signal strength distance is smaller than 200 dBm. The *k*-NN estimation approach was used with k=3. The indoor WiFi fingerprinting results are shown as yellow pins in Figure 10. Considering the good performance of GNSS in the outdoor environment, only the results of WiFi fingerprinting in the indoor environment are considered. Even though we set sampling rate of WiFi as 1 Hz, the real-world WiFi updating rate was less than 0.3 Hz. This might due to the restriction of the smartphone or the Android system.

The WiFi positioning results did not have long-term shifts, but were subject to short-term jumps. Due to the averaging calculation in the *k*-NN approach, not all the WiFi positioning results had the same position as the RPS stored in the DB. However, it is still clear that the WiFi positioning results might have similar shifts with DB, e.g., the right part of the WiFi positioning shifted to the north in Figure 10a because of the shifts in the proposed DB.

**Figure 11:**
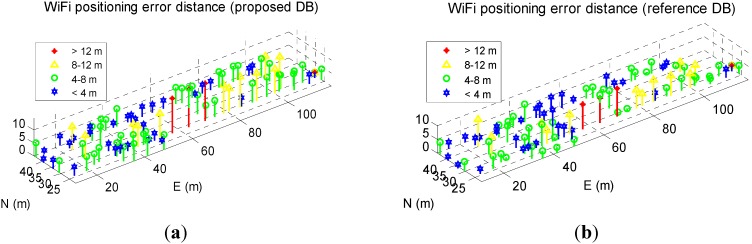
WiFi fingerprinting error distribution when using proposed DB (**a**) and using reference DB (**b**).

Figure 11a,b show the WiFi positioning errors on the navigation trajectory, where the *x*- and *y*-axis indicate the length of the trajectory in the west-east and south-north directions, and the *z*-axis is the positioning error distances.

The largest WiFi positioning errors existed in the middle area with abundant WiFi signals, instead of in the marginal areas with fewer WiFi signals, which does not meet our intuition. A possible reason for this is that the middle area has strongest signals, which causes ambiguity issue around this area. Integrating PDR with WiFi may probably mitigate the ambiguity issue, which is beyond the scope of this article.

**Figure 12:**
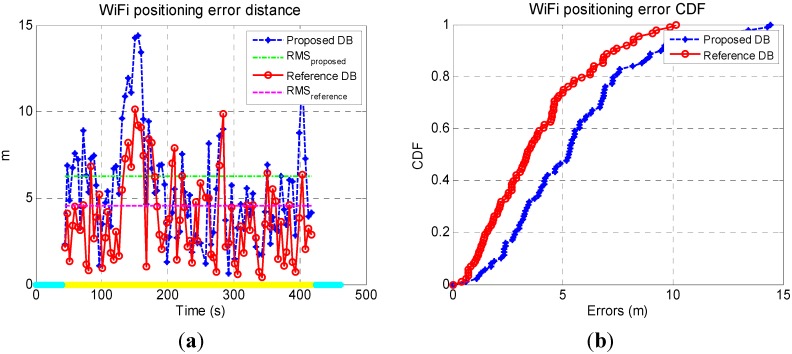
Time series of WiFi fingerprinting errors (**a**) and corresponding CDF (**b**).

To further evaluate the WiFi positioning errors, the time series of the errors are illustrated as blue dots and lines in Figure 12a. The RMS values of the errors are also calculated and shown as magenta lines. The yellow and blue lines on the x-axis indicate the indoor and outdoor environment, respectively. The statistical error CDF of both solutions are shown in Figure 12b.

With a relatively accurate DB (the reference DB), the max error and RMS error of WiFi positioning were 10.1 m and 4.6 m. These values increased to 14.2 m and 6.1 m when using the proposed DB. Therefore, using the proposed DB which had shifts with a RMS of 5.7 m, the final fingerprinting errors increased for 4.1 m in max value and 1.5 m in RMS. This indicated that there were other error sources in smartphone-based WiFi positioning which were more significant than such level of DB shifts. We can also see that the WiFi positioning results with both the proposed DB and the reference DB has large errors during 100 ~ 200 s, which indicates that such large errors may be introduced by the error sources in the WiFi positioning process, instead of the DB errors.

There is approximately 80% fewer errors than 7.5 m when using the proposed DB, while around 80% fewer errors than 6.0 m when using the reference DB. Therefore, the sample WiFi positioning results using the proposed DB were also 1.5 m worse than those using the conventional floor plan aided DB.

### 4.2. Tests at ENB

#### 4.2.1. Trajectory for Building DB

**Figure 13:**
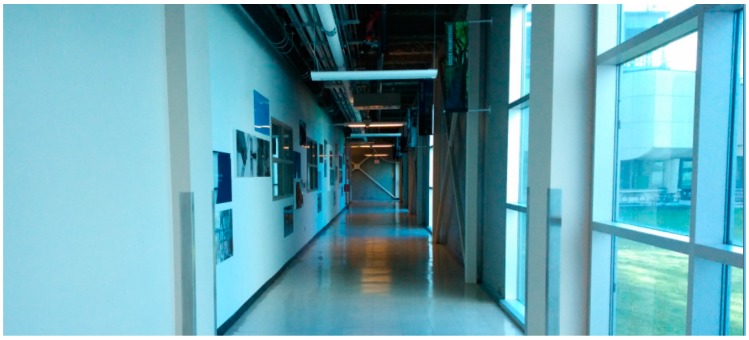
Indoor test environment at ENB.

ENB is mainly used for walking. Therefore, the environment at ENB is different from that at EEEL. Compared with EEEL, there are fewer WiFi APs and fewer metallic infrastructures at ENB. Figure 13 shows the indoor test environment. The test area at ENB was approximately 140 × 60 m^2^.

**Figure 14:**
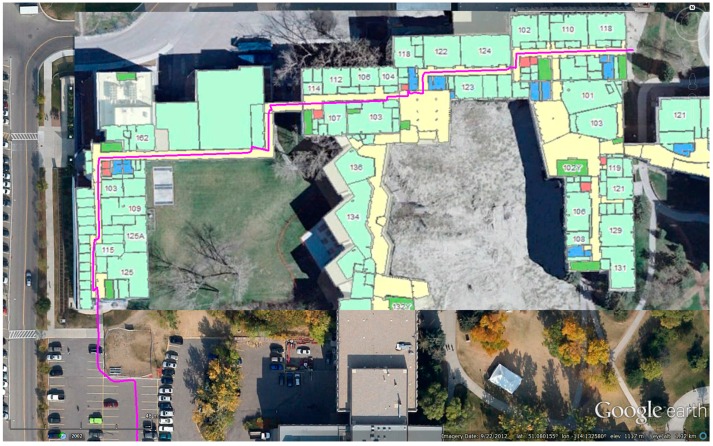
The trajectories used to generate WiFi DB in ENB.

The corridors inside ENB is long and narrow, which is different from those in EEEL. The test trajectory is shown in Figure 14.

#### 4.2.2. Building WiFi DB Using Sensor-Based Navigation Solution

**Figure 15:**
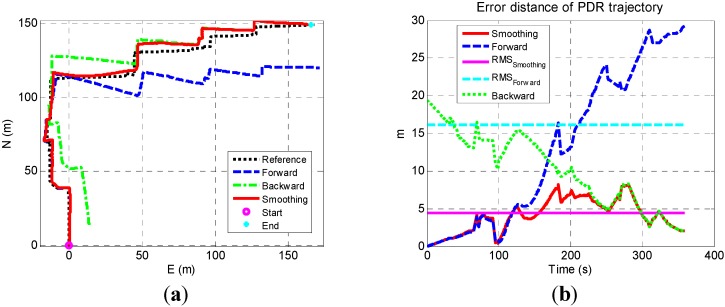
Sensor-based navigation solutions (**a**) and corresponding error distances (**b**).

The sensor-based navigation solutions are shown in Figure 15a. The blue dashed line, green dashed line, red solid line, and black dotted line are the results of forward, backward, smoothed solution, and the reference, respectively. The start and end points indicates the starting points of forward and backward PDR. The error distances of these solutions are illustrated in Figure 15b. The blue dashed line, green dotted line, and red solid line represent the error distances of forward, backward, and smoothed results, respectively. The magenta solid line and cyan dashed line indicate the RMS values of the forward the smoothed results.

Due to the straightforward and long shape of the ENB trajectory, the navigation error at the end of the forward and backward PDR reached 25 m and 20 m, which is larger than those in the EEEL tests. However, the max error and RMS error were reduced from 29.2 m and 15.9 m in forward results to 8.1 m and 3.9 m in smoothed results.

**Figure 16:**
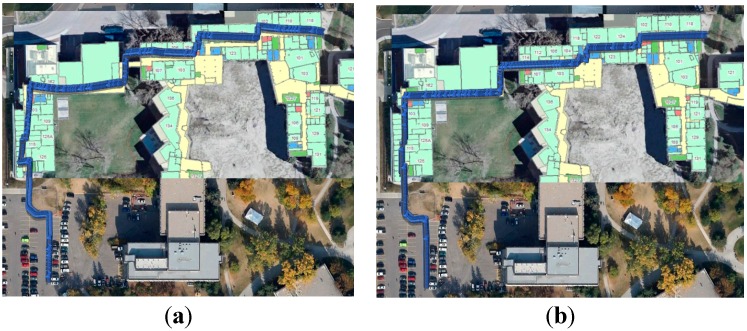
The DB built through proposed method (**a**), and the reference DB built by using the conventional floor plan aided training approach (**b**).

The smoothed navigation solutions were used to build the WiFi fingerprint DB, as shown in Figure 16a. The reference DB generated through the conventional floor plan aided method is shown in Figure 16b. It is clear that the proposed DB has some shifts in the middle part even after smoothing when comparing with the true floor plan.

**Figure 17:**
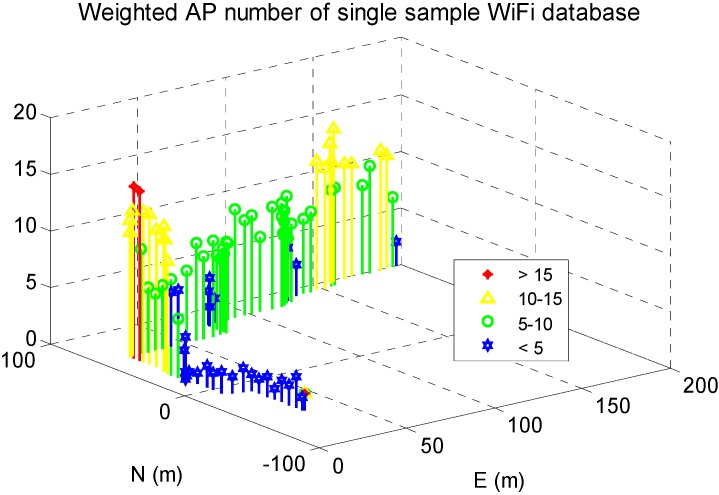
WiFi signal distribution in the reference DB.

The WiFi signal distribution in the DB was shown in Figure 17. There are generally many fewer WiFi signals on this trajectory than those in EEEL because the tests were conducted on the 0-th floor, which is mainly used for walking instead of working.

#### 4.2.3. WiFi Fingerprinting Using Generated DB

**Figure 18:**
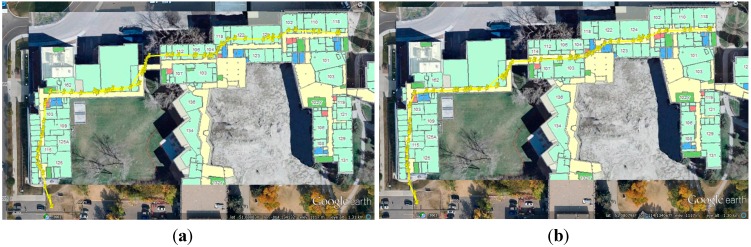
WiFi fingerprinting result using proposed DB (**a**) and that using reference DB (**b**).

In this WiFi positioning test, we use the same trajectory as the DB training tests, but walked in the opposite direction. The WiFi fingerprinting results with the proposed DB and the conventional DB were shown in Figure 18a,b respectively.

**Figure 19:**
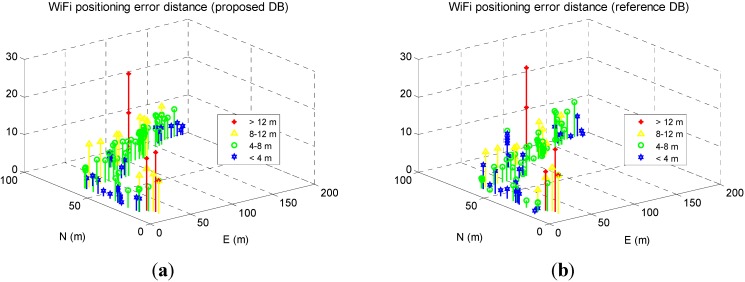
WiFi fingerprinting result using proposed DB (**a**) and that using reference DB (**b**).

Some part of the WiFi positioning results shifted to the north in Figure 18a because of the shifts in the proposed DB. Figure 19 a,b show the WiFi positioning errors on the navigation trajectory, where the *x*- and *y*-axis indicate the length of the trajectory in the west-east and south-north directions, and the *z*-axis is the positioning error distances.

**Figure 20:**
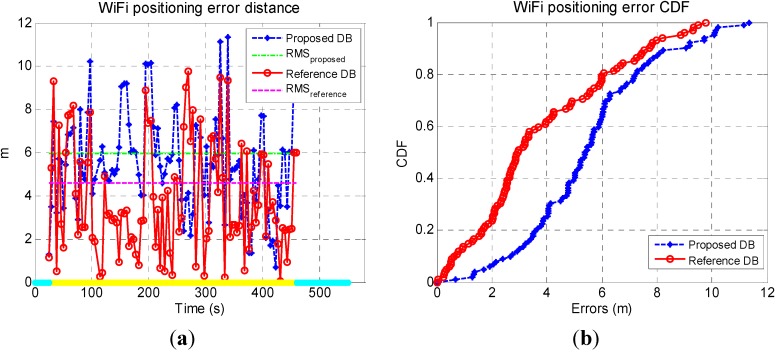
Time series of WiFi fingerprinting errors (**a**) and corresponding CDF (**b**).

Compared with Figure 17, the largest WiFi positioning errors existed in the areas with fewer WiFi signals. To further evaluate the WiFi positioning errors, Figure 20a illustrates the time series of the WiFi positioning errors and their RMS value are illustrated, and Figure 20b shows the statistical error CDF of both solutions.

The RMS of WiFi positioning errors decreased from 6.0 when using the proposed DB to 5.1 m when using the reference DB, while the max error increased from 11.4 m to 12.5 m. Therefore, even using the proposed DB which had shifts with a RMS of 3.9 m, the final WiFi fingerprinting results increased for only 0.9 m, which further verified that there were other error sources in smartphone-based WiFi positioning which is more significant than such level of DB shifts.

There are approximate 80% fewer errors than 7.1 m when using the proposed DB, while around 80% fewer errors than 6.4 m when using the conventional DB. Therefore, the sample WiFi positioning results using the proposed DB were 0.7 m worse than those using the conventional floor plan aided DB.

## 5. Conclusions

This paper presents a method which trains the WiFi fingerprint database using sensor-based navigation solutions. Since MEMS sensors provide only short-term accuracy but suffer from the accuracy degradation with time, we use a strategy that combine different navigation trajectories that move in and out of a building into the database, and restrict the time length of available indoor navigation trajectories to a certain time threshold (e.g., 10 min). This simple strategy can help control the sensor-based navigation errors. In addition, we conduct post-processing (smoothing) to improve the sensor-based navigation solution, since there are accurate GNSS position updates on the starting and ending points of indoor trajectories. Results show that the errors (RMS) of pedestrian navigation along different trajectories were reduced to 5.7 m during 5–10 min indoor walking.

Furthermore, we evaluated the effect of DB shifts on WiFi fingerprinting using the proposed DB (*i.e.*, the database generated using the proposed method). Results show that when the error of the proposed DB was 5.7 m, the WiFi positioning error was 6.1 m, which is 1.5 m larger than that with the reference DB (*i.e.*, the database built through the floor-plan aided method). In the tests inside another building, when the error of the proposed DB was 3.9 m, the WiFi positioning error was 6.0 m, which is 0.9 m larger than that with the reference DB. Therefore, the difference between the WiFi fingerprinting results with the proposed DB and the reference DB was not significant when comparing with the WiFi fingerprinting errors with smartphones. Therefore, although constructing a high-quality DB is vital as fingerprinting accuracy highly depend on DB quality, the smartphone-based fingerprinting errors will not increase linearly according to DB errors because there are other error sources.

Future works will focus on researching WiFi positioning based on crowd-sourcing, where the database accuracy can be further improved with mass data of sensor-based navigation.
